# *Hordeum murinum* aspiration revealed by a pneumopleurocutaneous fistula in a 15-month-old infant

**DOI:** 10.1186/s12887-021-03016-0

**Published:** 2021-12-05

**Authors:** Nicolas Richard, Audrey Paygambar, Hubert Ducou Le Pointe, Sarah Biaz, Harriet Corvol

**Affiliations:** 1 Pediatric Pulmonology Department, Assistance Publique – Hôpitaux de Paris (AP-HP), Hôpital Trousseau, Sorbonne Université, 26 avenue du docteur Netter, 75012 Paris, France; 2 Centre de Recherche Saint-Antoine (CRSA), Inserm UMR_S938, Paris, France; 3 Department of Visceral and Neonatal Paediatric Surgery, AP-HP, Hôpital Trousseau, Sorbonne Université, Paris, France; 4 Pediatric Radiology Department, APHP Hôpital Trousseau, Sorbonne Université, Paris, France; 5 Pediatric Department, Sainte Camille Hospital, Bry sur Marne, France

**Keywords:** Child, Pulmonology, Foreign body aspiration, Case report

## Abstract

**Background:**

*Hordeum murinum* is a specie of grass rarely reported among the aspirated foreign body. It has high tissue penetration power and may cause lung damages.

**Case presentation:**

We report the case of a 15-month-old girl who choke while playing in the grass without any evident cause. This episode was immediately followed by vomiting and coughing with traces of blood. While she was fine during the following week, she relapsed at day (D) 7 with fever. At D10, she was finally hospitalized for signs of respiratory distress. The chest CT-scan revealed a voluminous right sub pleural empyema with an aerial component, responsible for the collapse of the right lower lobe, and complicated by a pneumopleurocutaneous fistula to the right paravertebral muscles. Intravenous antibiotics were prescribed, but no invasive procedure was performed. At D18, the spikelet of a false barley spontaneously externalized through the fistula. Evolution was favorable thereafter with disappearance of the fever and progressive decrease of the biological inflammatory syndrome. The follow-up at 4 months was reassuring, with normal clinical evaluation, and complete regression of the empyema on the chest X-rays.

**Conclusions:**

*Hordeum murinum* is a rare type of foreign body, and the aspiration often goes unnoticed. In these peculiar cases, CT-scans can be as informative as bronchoscopies, and the evolution is usually favorable after fistulization.

## Background

Foreign body aspiration is a frequent reason for emergency consultation in pediatrics. It happens mostly in children before 3 years old, and can lead to serious morbidity and mortality. It is often followed by suffocation, acute cough, and sometimes respiratory distress and cyanosis. The classical symptoms triad is sudden onset cough, followed by persistent cough and wheezing [1]. The exact moment when the foreign body is aspired may be unnoticed by the parents. A chest X-ray is needed in addition to clinical symptoms and physical examination to decide the necessity of a bronchoscopy. The nature of the foreign body may be diverse, such as food, plant, piece of plastic, mineral, animal or chemical compounds, etc. *Hordeum murinum* is a quite widespread and common specie of grass commonly known as wall barley or false barley. While it is rarely reported among the aspirated foreign body, it has high tissue penetration power and may cause lung damages [2].

## Case presentation

The mother of a 15-month-old girl saw her choke while playing in the grass in a public park without any evident cause. This infant was previously healthy with the exception of one bronchiolitis at 7 months old. The choking episode was immediately followed by coughing and vomiting with traces of blood. She visited the emergency department, but was send back home as the clinical evaluation was normal.

While she was fine during the following week, she relapsed at day (D) 7 with vomiting, coughing and fever. She returned to the emergency department at D10 where the clinical evaluation showed signs of respiratory distress (respiratory rate: 40 pm; oxygen saturation: 98% in room air) associated with fever, and she was hospitalized. Significant laboratory findings included an elevated C-reactive protein (CRP) 144 mg/L and leukocytosis (white blood cell count 26.0 × 10^9^/L, with neutrophil count 22.0 × 10^9^/L). The RT-PCR for SARS-CoV-2 in nasopharyngeal swab and stool, as well as the tuberculin skin test were negative. The chest X-rays revealed a right round hilar opacity.

**Fig. 1:**
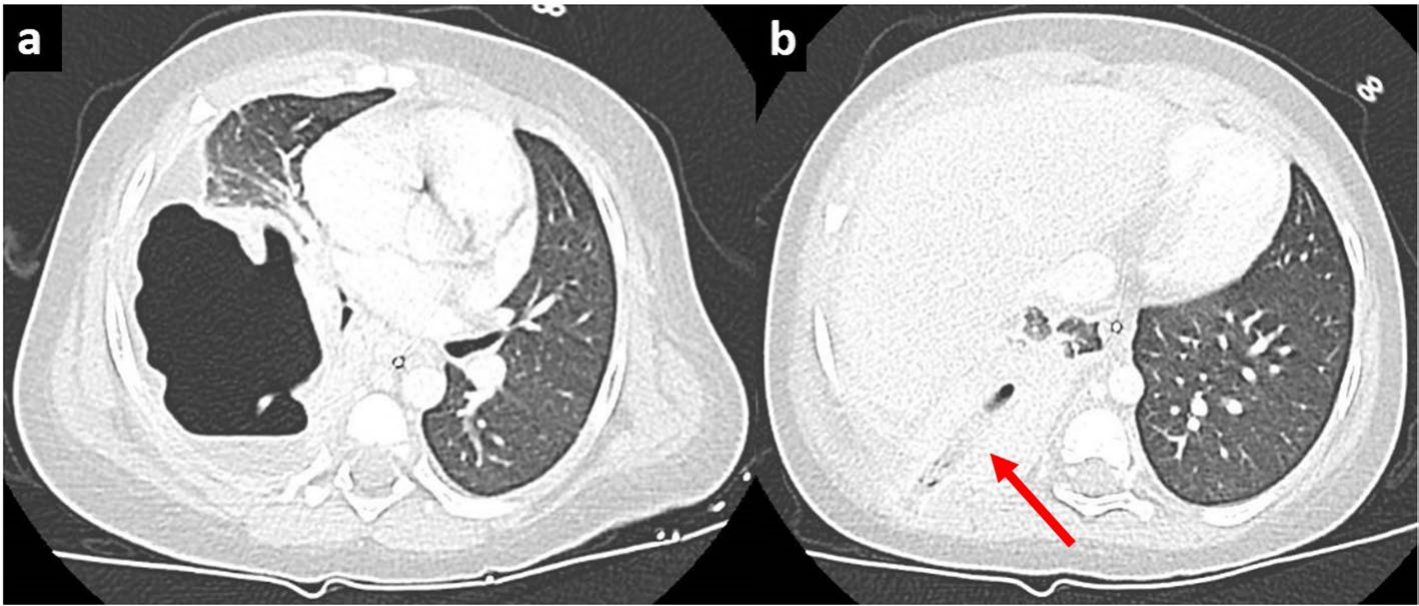
CT-scan at day 12 shows a voluminous right sub pleural empyema with an aerial component (**1a**), responsible for the collapse of the right lower lobe, and complicated by a pneumopleurocutaneous fistula to the right paravertebral muscles (red arrow, **1b**)

Although oral amoxicillin was started at D10, fever persisted and blood inflammation continued to increase, with the CRP reaching 395 mg/L at D12. A chest CT-scan was then performed revealing a voluminous right sub pleural empyema with an aerial component (Fig. 1a), responsible for the collapse of the right lower lobe, and complicated by a pneumopleurocutaneous fistula to the right paravertebral muscles (Fig. 1b). The antibiotic regimen was changed to intravenous cefotaxim associated with vancomycin and clindamycin, as recommended by the French guidelines [3].

**Fig. 2:**
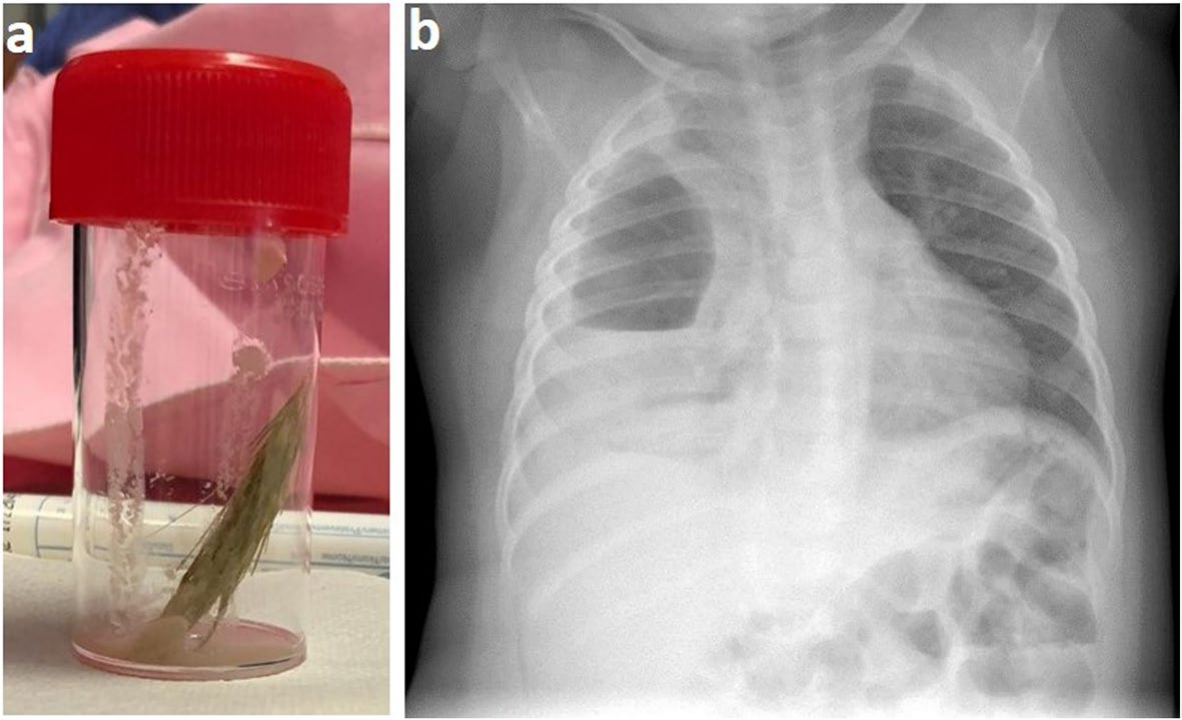
After the externalization: picture of the aspired wheat spikelet (**2a**); the chest X-ray shows a right pleural effusion (**2b**)

At D18, the spikelet of a false barley (shown in Fig. 2a) was externalized through the fistula; and the chest X-rays showed a right pleural effusion (Fig. 2b). The diagnostic of aspiration of the spikelet of a *Hordeum murinum* complicated by a pleural empyema, and revealed by a pneumopleurocutaneous fistula to the right paravertebral muscles was then ascertained.

**Fig. 3:**
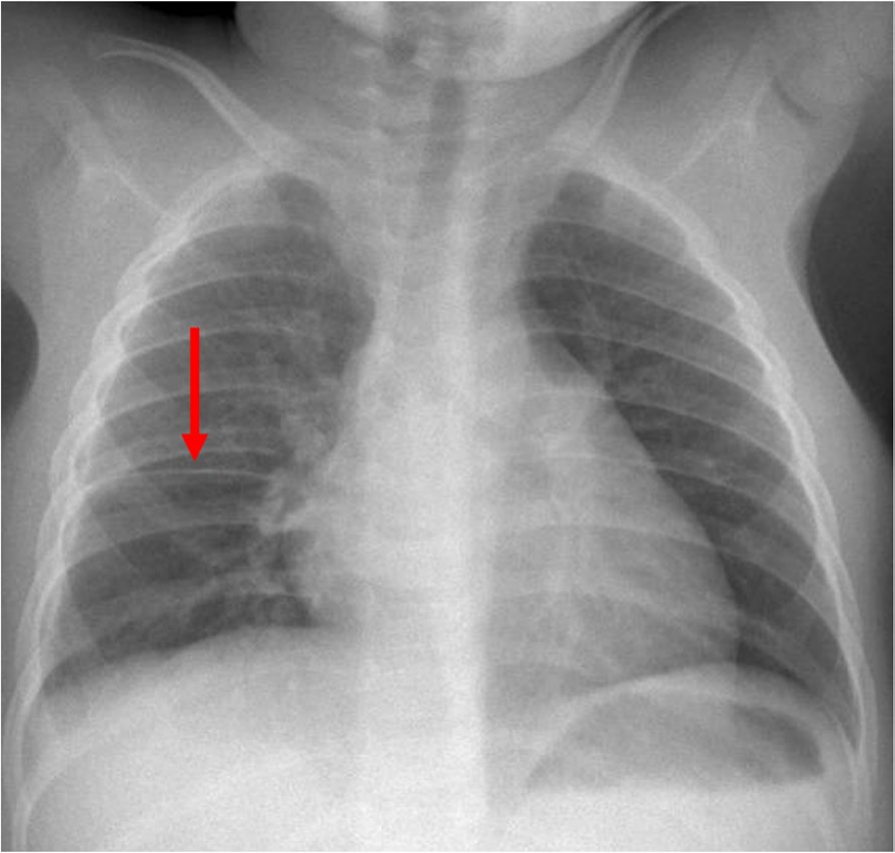
Normal chest X-ray 3 months after the spikelet aspiration, with only a slight pleural fissure thickening (red arrow)

Bacteriologic analysis of the wheat spikelet revealed *Haemophilus sp*., Methicillin-Susceptible *Staphyloccocus aureus* (MSSA) and *Streprococcus anginosus*. Antibiotics were continued, associated with wet bandages at the fistulization site. Evolution was favorable thereafter with disappearance of the fever and progressive decrease of the biological inflammatory syndrome. At D21, antibiotics were changed to amoxicillin/clavulanic acid intravenously during 10 days, followed by 15 days orally. She was discharged home after 1 month of hospitalization with a significant improvement of the chest X-ray. The follow-up 3 months after the grass aspiration was reassuring, with normal clinical evaluation and complete regression of the empyema on the chest X-ray (Fig. 3).

## Discussion and conclusions

This case is remarkable because of the pneumopleurocutaneous fistula to the right paravertebral muscles followed by the externalization of the *Hordeum murinum* spikelet. The clinical presentation was usual at the beginning. First, the age of the child (15 months old) was consistent with those reported in other studies where aspirations occur mostly before 3 years old [1, 4, 5]. Clinical symptoms were also common, associating cough and fever [4]. When such symptoms appear after a history of witnessed choking, a chest X-ray is required, and shows abnormalities in about one third of the cases: radiopaque foreign body, unilateral reduced air entry, infection, etc. [6]. A bronchoscopy could also be necessary, depending on the radiological findings. In our case, no bronchoscopy was performed as the CT-scan showed that the foreign body was localized in the right part of the bronchial tree. Besides, the anatomical structure of the right main bronchi makes foreign bodies more likely to be incarcerated [7].

The type of foreign body varies in proportion across countries. While Sink et al. reported more than 50% of food pieces in the US [1], Zhong et al. found a large majority of plants in China [5]. *Hordeum murinum* is the principal foreign body reported to lead to chest cutaneous fistula. It was described in 3 other cases in children of 5, 12 and 13 years-old, so older than our case [2, 8, 9]. The duration between the foreign body aspiration and the fistulization is variable, from a couple of weeks, such as this case, to several months [8]. It could be revealed by different symptoms: hemoptysis, lung abscess, abdominal pain, bronchiectasis, and spontaneous fistulization [10]. A bronchoscopy is frequently performed, but does not systematically find the foreign body; whereas CT scans show a pleural effusion and/or the foreign body like in our patient. Antibiotics were prescribed in each case, but it seems that it is mostly the spontaneous fistula that led to the spectacular clinical improvement and quick discharge. As in our case, follow-ups show normalization of the chest X rays and complete healing. Other cases of unextractable bronchial foreign body can necessitate surgery and be associated with irreversible damage of lung tissue [11].

To conclude, this case illustrates an atypical spontaneous pneumopleurocutaneous fistula of the spikelet of a *Hordeum murinum* following its aspiration in an infant. This type of foreign body is rare, and the aspiration often goes unnoticed. In these peculiar cases, CT-scans can be as informative as bronchoscopies, and the evolution is usually favorable after the fistulization.

## Data Availability

Not applicable.

## Abbreviations

CRP: C-reactive protein
CT-scans: Computed tomography scan
